# Regulation and expression of multidrug resistance (MDR) transcripts in the intestinal epithelium

**DOI:** 10.1038/sj.bjc.6690475

**Published:** 1999-06

**Authors:** M Li, R Hurren, R L Zastawny, V Ling, R N Buick

**Affiliations:** Ontario Cancer Institute/Princess Margaret Hospital and Department of Medical Biophysics, University of Toronto, Toronto, Ontario, M5G 2M9, Canada; Allelix Biopharmaceutical Inc., Missanga, Ontario, L4V 1V7, Canada; BC Cancer Research Center, Vancouver, BC, V5Z 1L3, Canada

**Keywords:** intestine, gene expression, differentiation, mdr1a, mdr1b

## Abstract

A paucity of information exists on the regulation of gene expression in the undifferentiated intestine. The intestinal epithelium is one of the few normal tissues expressing the multidrug resistance (MDR) genes that confer the multidrug resistant phenotype to a variety of tumours. Expression of mdr1a has been observed in the primitive rat intestinal epithelial cell line, IEC-18. It is hypothesized that characterization of MDR gene expression in IEC-18 cells will provide insight into gene regulation in undifferentiated intestinal cells. A series of hamster mdr1a promoter deletion constructs was studied in IEC-18 and a region with 12–13-fold enhancer activity was identified. This region was shown to function in an orientation- and promoter context-independent manner, specifically in IEC-18 cells. Unexpectedly, Northern probing revealed a greater expression of mdr1b than mdr1a in IEC-18 cells. A quantitative reverse transcription polymerase chain reaction assay was used to compare the relative expression of MDR genes in IEC cells, fetal intestine, and in the undifferentiated and differentiated components of adult intestinal epithelium. MDR transcript levels in IEC cells were found to resemble those of fetal intestine and small intestinal crypts, where a conversion from mixed mdr1a/mdr1b to predominantly mdr1a expression occurs as cells mature. This work describes two contributions to the field of gene regulation in the undifferentiated intestine – first, the initial characterization of a putative mdr1a enhancer region with specificity for primitive intestinal cells and secondly, the first report of mdr1b detection in the intestine and its expression in primitive cell types. © 1999 Cancer Research Campaign


British Journal of Cancer (1999) 80(8), 1123–1131
© 1999 Cancer Research Campaign
Article no. bjoc.1999.0475

### Regulation and expression of multidrug resistance (MDR) transcripts in the intestinal epithelium

**M Li[1], R Hurren[1], RL Zastawny[2], V Ling[3] and RN Buick***

[1]Ontario Cancer Institute/Princess Margaret Hospital and Department of Medical Biophysics, University of Toronto, 610 University Avenue, Toronto, Ontario, Canada M5G 2M9; [2]Allelix Biopharmaceutical Inc., Missanga, Ontario, Canada L4V 1V7; [3]BC Cancer Research Center, Vancouver, BC, Canada V5Z 1L3

**Summary** A paucity of information exists on the regulation of gene expression in the undifferentiated intestine. The intestinal epithelium is one of the few normal tissues expressing the multidrug resistance (MDR) genes that confer the multidrug resistant phenotype to a variety of tumours. Expression of mdr1a has been observed in the primitive rat intestinal epithelial cell line, IEC-18. It is hypothesized that characterization of MDR gene expression in IEC-18 cells will provide insight into gene regulation in undifferentiated intestinal cells. A series of hamster mdr1a promoter deletion constructs was studied in IEC-18 and a region with 12–13-fold enhancer activity was identified. This region was shown to function in an orientation- and promoter context-independent manner, specifically in IEC-18 cells. Unexpectedly, Northern probing revealed a greater expression of mdr1b than mdr1a in IEC-18 cells. A quantitative reverse transcription polymerase chain reaction assay was used to compare the relative expression of MDR genes in IEC cells, fetal intestine, and in the undifferentiated and differentiated components of adult intestinal epithelium. MDR transcript levels in IEC cells were found to resemble those of fetal intestine and small intestinal crypts, where a conversion from mixed mdr1a/mdr1b to predominantly mdr1a expression occurs as cells mature. This work describes two contributions to the field of gene regulation in the undifferentiated intestine – first, the initial characterization of a putative mdr1a enhancer region with specificity for primitive intestinal cells and secondly, the first report of mdr1b detection in the intestine and its expression in primitive cell types.

**Keywords**: intestine; gene expression; differentiation; mdr1a; mdr1b

The epithelial lining of the mammalian intestinal mucosa is an excellent tissue for studying molecular aspects of development, differentiation and tumorigenesis. Morphogenesis from fetal endoderm to the adult structure occurs along strict temporal and spatial gradients during the last 5 days of rodent gestation (Trier and Moxey, 1979; Deren, 1987). The adult intestinal mucosa is organized as a single-layered epithelium lining crypts, which invaginate below the surface of the gut and villi, which protrude into the gut lumen. Cells lining the villi are continually being replaced by the proliferation, differentiation and migration of stem cells within the crypts (Potten and Loeffler, 1990). Thus, the crypt and villus structures represent undifferentiated and differentiated cellular compartments respectively. The expression patterns of numerous genes have demonstrated that differential gene expression occurs in two dimensions – along the crypt–villus axis and along the proximal–distal axis (Asp et al, 1975; Traber, 1990; Trezise and Buchwald, 1991). Thus, geographic and cell-specific patterns of gene expression are established during a period of rapid cellular proliferation, differentiation and morphogenesis, and maintained despite continual cell migration and renewal.

The transcriptional regulation of an increasing number of intestinal genes have been described and from this work intestine-specific promoter elements and transcription factors are beginning to emerge (Cohn et al, 1992; Suh et al, 1994; Traber, 1994). Molecular research in the intestine has so far focused on transcriptional regulation in mature, fully differentiated intestinal cell types. To our knowledge no promoter elements have been identified which function in developing or undifferentiated intestinal epithelial cells. To date, the regulation of only three genes which are expressed in undifferentiated intestine have been reported: the murine homeobox gene *cdx-1* (Hu et al, 1993), the cystic fibrosis transmembrane conductance regulator (CFTR) (Koh et al, 1993; McDonald et al, 1995) and the fetal serum protein α-fetoprotein (AFP) (Tyner et al, 1990; Cirillo et al, 1995). In each of these cases, analysis was either performed in colonic carcinoma cell lines or focused on elements which repress expression in mature enterocytes. Since transcripts which function in development, lineage determination and tumorigenesis might be expected to first appear in crypt or fetal intestinal cells, promoter elements which regulate transcription in undifferentiated intestinal cells would be of interest.

IEC cells are a series of spontaneously immortalized crypt-like cell lines from fetal and newborn rat small intestine (Quaroni et al, 1979; Quaroni and Isselbacher, 1981; Quaroni, 1985). In the IEC-18 cell line, expression of the multidrug resistance (MDR) gene, mdr1a, has previously been observed (KL Duechars, unpublished data). MDR genes encode P-glycoprotein, a transmembrane efflux pump which mediates the multidrug resistant phenotype in a variety of tumours (for reviews see Gottesman and Pastan, 1993; Gottesman et al, 1995, 1996). Three classes of MDR genes have been defined. Only classes I and II can confer drug resistance, although their physiological substrates remain unidentified; class

*Received 22 April 1998*
*Revised 6 January 1999*
*Accepted 7 January 1999*

*Correspondence to:* R Hurren

*Deceased July 1996

III functions in the liver to transport phosphatidylcholine into bile (Ruetz and Gros, 1994). Humans have only class I and III genes (MDR1 and MDR3/MDR2), while rodents express all three classes – mdr1a/mdr3, mdr1b/mdr1 and mdr2 in mouse; pgp1, pgp2 and pgp3 in hamster; mdr1a/pgp1, mdr1b/pgp2 and mdr2/pgp3 in rat. Each family member exhibits distinct patterns of tissue-specific expression with the intestinal epithelium being one of the few normal tissues known to express high levels of mdr1a (Gatmaitan and Arias, 1993). Transcriptional regulation of multiple family members, from many species, has been studied extensively (Thorgeirsson et al, 1994; Smit et al, 1995). However, very few cell type-specific promoter elements have been described in MDR genes, the only published example being a hepatoma cell-specific enhancer in the mouse mdr1b promoter (Song et al, 1995).

It is hypothesized that insight into gene regulation in undifferentiated intestinal cells can be obtained through study of MDR gene expression in IEC-18 cells as a model system. First, to identify promoter elements functioning in primitive intestinal cell types, the hamster pgp1 promoter was mapped in IEC-18 cells. Second, employing a quantitative reverse transcription polymerase chain reaction (RT-PCR) comparative analysis to validate the use of IEC-18 cells as a model, novel gene-specific expression patterns of MDR family members were identified in undifferentiated intestinal cell types.

### MATERIALS AND METHODS

### Recombinant plasmids and oligonucleotides

Generation of pgp1 promoter and deletion constructs were described in Zastawny and Ling (1993). Enhancer constructs p100/25CAT and p25/100CAT were constructed by blunt-ended cloning of an *Xba*1(–100)/*Hpa*1(–25) fragment from the pgp1 promoter into the *Bgl* II site of pCAT-Promoter (Promega) in the forward and reverse orientations respectively. PCR primers for rat ribosomal protein L32, 5′ (AGAGGCATCGACAACAGGGTG) and 3′ (CTATTCATTCTCTTCGCTGCG), result in a 294-bp product (Rajchel et al, 1988). MDR gene-specific primer pairs were selected from the 3′ untranslated regions of the three rat mdr genes (Deuchars et al, 1992). Sequences for the primers used were: mdr1a – 5′ (CTGTGACCATGCGAGATG) and 3′ (GGTGCTAGGGATCTGGAC), mdr1b – 5′ (TATTTGAGGTGCTAAGTATTTC) and 3′ (GTGCGGAAAGGCATCCCA), mdr2–5′ (AACTTATGAACTTGTTACAGTA) and 3′(CCAAATGTCCATGGAAG).

### Cell culture, transient transfection and CAT assays

RC3 (rat hepatoma) cells were maintained in SWIM S77 medium supplemented with 4 mM glutamine, 20% horse serum and 5% fetal bovine serum (FBS). AuxB1 (Chinese ovary), NRK-52E (rat kidney) and SW620 (human colon carcinoma) lines were carried in α-MEM plus 10% FBS. IEC cells were grown in α-MEM supplemented with 20 mM glucose, 2 mM glutamine, 0.27 units $ml^{-1}$ insulin and 5% FBS. Transient transfections of RC3, AuxB1 and SW620 were performed with lipofectAMINE reagent (GibcoBRL) as directed by the manufacturer. IEC-18 cells ($10^7$ cells in 100 μL of HEPES-buffered saline) were electroporated in 0.4-cm gap cuvettes with one pulse at 450 V, 125 μFd (BioRad Gene Pulser). In all cases pCMV–β-gal was co-transfected as an internal control for transfection efficiency. CAT (phase-extraction method) and β-gal assays were performed 48 h after transfection (1993) and data were represented as the mean and standard error of 3–4 independent trials where CAT activity was normalized to β-gal.

### RNA isolation

Small intestinal crypts, villi and colonic intestinal crypts were isolated from 8-week-old male CD rats by incubation in 30 mM EDTA at 37°C and mechanical vibration of everted intestinal segments following previously established methods (Cano-Gauci et al, 1993) with the following modifications:

1. All tissue samples were isolated in calcium- and magnesium-free phosphate-buffered saline without fixation.
2. Villi, each containing approximately 3500 cells, were released within the first 5 min of incubation in EDTA and confirmed to be free of crypts under light microscopy.
3. Mixed villus and crypt populations released over the next 20 min by multiple rounds of vibration into fresh solutions were discarded.
4. Single crypts, containing roughly 250 cells each, released after 30 min of EDTA incubation were examined under light microscopy, and individually selected under suction with drawn-out, bent pasteur pipettes.

Pooled populations of crypts and villi were isolated from the duodenum, jejunum, ileum and colon. RNA was prepared from all samples immediately after isolation with 1 ml of TRIZOL reagent (GibcoBRL), according to manufacturer's specification. RNA from cultured cell lines and whole tissue was isolated directly with the TRIZOL reagent. Fetal intestinal RNA at various stages of development was prepared from whole intestine of CD rat embryos, pulverized in liquid nitrogen with a mortar and pestle, using the acid-guanidinium thiocyanate method of Chomczynski and Sacchi (1987).

### Northern blot analysis

For Northern blot analysis, 5 μg of RNA were separated by electrophoresis on 0.75% agarose-formaldehyde gels in phosphate buffer and transferred to Zetaprobe membranes (Bio-Rad Labs) in 10 × saline–sodium citrate (SSC). Filters were prehydridized at 42°C in 45% formamide, 5 × Denhardt's, 2% sodium dodecyl sulphate (SDS), 5 × Saline–Sodium phosphate–EDTA (SSPE) and 100 μg $ml^{-1}$ each of salmon sperm DNA and poly-$A^+$. Hybridization was performed under the same conditions using random-primed, $^{32}P$-labelled cDNA probes for mouse L32 (Dudov and Perry, 1984) or the 2B13-155 probe which recognizes rat MDR genes (generously provided by JA Silverman). Filters were washed in 2 × SSC, 0.2% SDS for 30 min at 42°C, followed by 30 min wash in 1 × SSC and 0.1% SDS at 65°C before exposure to a phosphorimager screen and quantification with ImageQuant software (Molecular Dynamics).

### RT-PCR analysis

Reverse transcription was performed with 1 μg of DNAase-treated RNA for 1 h at 37°C with 25 pmol of a dT24 synthetic oligonucleotide, 12 units AMV reverse transcriptase (Boehringer Mannheim) in the supplied incubation buffer, 36 units RNAGuard (Pharmacia) and 1 mM each of dATP, dGTP, dCTP and dTTP

© 1999 Cancer Research Campaign

(dNTPs). For each MDR gene, 5 μg of the resulting cDNA were amplified in 10 mM Tris pH 8.3, 1.5 mM magnesium chloride, 50 mM potassium chloride, 0.1% Triton X-100, 0.2 mM each of dNTPs, 25 pmol each of 5′ and 3′ primers and 2 units of *Taq* polymerase (Perkin-Elmer Cetus) with a final reaction volume of 50 μL, overlaid with an equal volume of mineral oil. Amplification was performed for 30 sequential cycles at 95°C for 30 s, 55°C for 30 s, 72°C for 30 s, followed by a final 10-min extension at 72°C. Equal aliquots of each PCR reaction were separated on a 2% agarose gel and photographed following ethidium bromide staining.

### Quantitative RT-PCR

Quantitative RT-PCR was performed as described in Murphy et al (1990). Wherever possible, reaction mixes were used to increase consistency. Following reverse transcription as described above, serial 1:2 dilutions of cDNA were made starting with the amount of cDNA empirically determined to permit amplification within the linear range of the gene of interest. Thus, serial dilutions were initiated with the following amounts of cDNA, expressed as ng of RNA in the cDNA reaction: for mdr1a – 10 ng from small intestinal crypts and villi, 50 ng from colonic crypts, 100 ng from fetal intestine and IEC-18 cells; for mdr1b – 100 ng from small intestinal crypts, fetal intestine and IEC-18 cells; for L32 – 100 pg from colonic crypts, fetal intestine and IEC-18 cells, 1 ng from small intestinal villi and crypts. Dilutions were amplified as described above except for the inclusion of 2 μCi ($^{32}$P)dCTP in each reaction. For each sample, eight serial 1:2 dilutions were amplified and 20 μl of PCR product were run on 5% acrylamide gels which were subsequently dried and exposed to a phosphorimager screen for 16 h. Quantification of gel bands was performed with ImageQuant software (Molecular Dynamics). For each sample, amplification of mdr1a, mdr1b and L32 was performed at the same time, independently amplified from the same cDNA reaction, using the same amount of specific radioactivity and exposed to the phosphorimager screen together. To determine the relative amounts of a gene expressed between two samples, the phosphorimaging counts are compared at the same amount of input cDNA, within the exponential range of each gene. MDR counts are normalized against similarly analysed L32 levels, corrected for tissue abundance.

### RESULTS

### Deletion mapping of the pgp1 promoter in IEC-18 cells

In humans, only the MDR class I transcript has been associated with expression in the intestinal epithelium and multidrug resistance in tumours. As a candidate gene in which to identify intestine-specific promoter elements, a series of previously characterized hamster MDR class I promoter deletion constructs (Zastawny and Ling, 1993) were studied in IEC-18 cells. Although IEC-18 is a rat cell line, the hamster MDR promoter was employed in this study because the rat mdr1a promoter has not yet been cloned. MDR promoters are highly conserved across rodent species among classes (Zastawny and Ling, 1993), the rat mdr1b promoter (Silverman and Hill, 1995) having 73% homology to hamster pgp2 and only 47% similarity to hamster pgp1. Although the cross-species promoter analysis used in this study may limit the applicability of the findings to MDR gene regulation, any

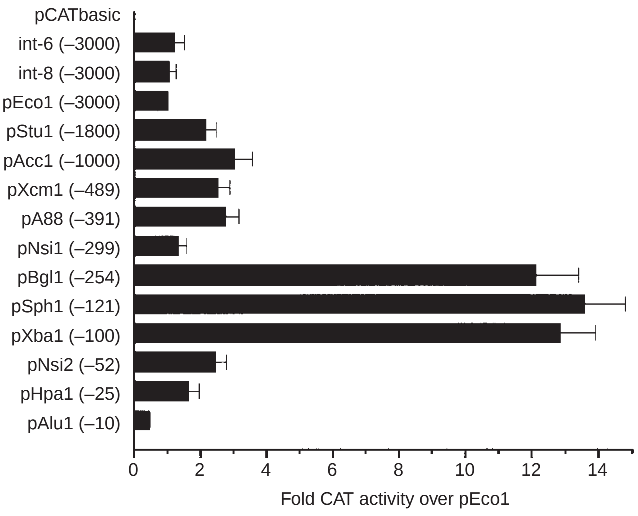

**Figure 1** Activity of pgp1 promoter-CAT deletion constructs in IEC-18. Deletion constructs of the hamster pgp1 promoter region driving a CAT reporter gene were transiently transfected into IEC-18 along with a CMV–β-gal plasmid by electroporation. CAT activity, normalized to β-galactosidase levels, are expressed relative to the largest construct, pEco1. The constructs int-6 and int-8 are identical to pEco1 with the addition of intron 1 seqences in the forward and reverse orientations respectively identified regulatory regions will still reflect promoter elements which function in intestinal gene expression.

Deletion constructs of the hamster pgp1 promoter region from –3000 to –10 bp (relative to the transcription start site) driving a CAT reporter gene, were transiently transfected into IEC-18 (Figure 1). CAT activity, normalized to co-transfected CMV–β-gal, is expressed relative to the largest construct, pEco1. Deletion from –3000 to –299 has no significant effect on promoter activity. A 13-fold increase in CAT activity is seen between pNsi1 and pBgl1, suggesting the deletion of a repressor element between –299 and –254. Further deletion from –100 to –52 results in a 12-fold drop in reporter activity, indicating the presence of an enhancer region. Core promoter activity is maintained up to –25, with further deletion significantly reducing the reporter expression. Previously published data demonstrate the repressor and enhancer regions identified here, do not exhibit the same activity in AuxB1 (Zastawny and Ling, 1993), and those findings were reproduced in the present study (data not shown). Similarly, intron 1 sequences which were shown to have enhancer activity in AuxB1 (plasmids int-6 and int-8), do not function in IEC-18 (Figure 1).

To further define and demonstrate the tissue-specificity of the potential repressor and enhancer elements, these regions were subcloned upstream of a heterologous promoter. Two repressor constructs were made by subcloning the regions between –299 to –254 and from –391 to –254, in both orientations, in front of the enhancerless SV40 promoter in the pCATpromoter vector. These constructs failed to demonstrate repressor activity in the forward orientation and while the reverse orientation abolished reporter activity, this effect was shown not to be cell type-specific (data not shown).

The potential enhancer region of the pgp1 promoter from –100 to –25 was subcloned into pCATpromoter in the forward (p100/25 CAT) and reverse (p25/100 CAT) orientations. In SW620, RC3

© 1999 Cancer Research Campaign 

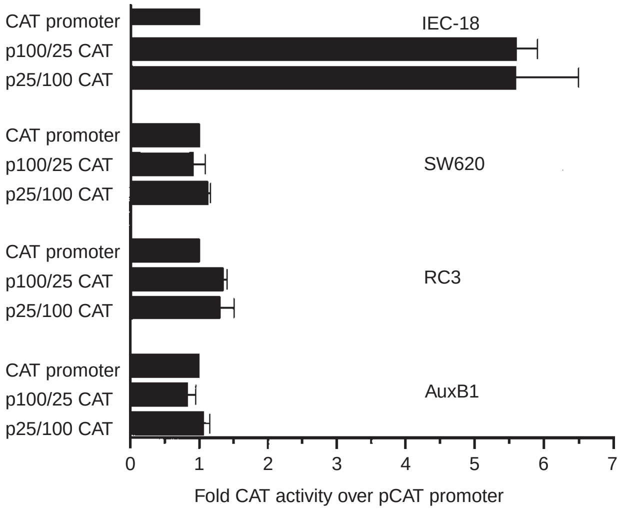

**Figure 2** Tissue-specificity of the pgp1 enhancer region. The –100 to –25 region of the pgp1 promoter was cloned upstream of the SV-40 promoter in the pCAT promoter vector in both forward and reverse orientations (p100/25 CAT and p25/100 CAT, respectively). Activity of these constructs was analysed in various MDR expressing cell lines, normalized to CMV-βgal, and expressed relative to the enhancer-less pCAT promoter and AuxB1 cells these constructs have the same activity as pCATpromoter (Figure 2). In contrast, transfection of these constructs into IEC-18 resulted in an increase in reporter activity over that of pCATpromoter. Thus, the –100 to –25 region of the pgp1 promoter contains an enhancer activity which functions in an orientation- and promoter context-independent manner, specifically in IEC-18 cells.

### Quantitative RT-PCR of MDR expression in the intestine

Surprisingly, transcript analysis in IEC-18 cells revealed a greater expression of mdr1b than mdr1a. Figure 3A depicts a Northern blot of IEC-18 RNA probed with 2B13-155, an mdr1b cDNA fragment which recognizes all three classes of MDR genes (Silverman et al, 1991). The 5.2- and 4.4-kb bands have previously been shown to correspond to mdr1a and mdr1b, respectively (Schrenk et al, 1992), and these transcript species were confirmed by probing with gene-specific oligonucleotides derived from the divergent 3′UTRs of each rat gene (Deuchars et al, 1992) (data not shown). In order to more fully explore the nature of MDR gene expression in IEC-18 cells, a quantitative RT/PCR assay was established to compare MDR gene-specific expression between intestinal tissue taken from fetal intestine, and along the proximal–distal and crypt–villus intestinal axes.

Initially, RT-PCR analysis was performed in various cell lines and intestinal tissues by amplification of mdr1a, mdr1b and mdr2 using equal amounts of a single cDNA synthesis reaction (Figure 3B). Contamination of RNA samples with genomic DNA was ruled out by amplification of RNA without reverse transcription and gene-specificity of the PCR primers was demonstrated by amplification of MDR genes against each MDR genomic DNA template (Deuchars et al, 1992) (not shown). The observed sizes of the major PCR product for mdr1a, mdr1b and mdr2 are consistent with their expected 555 bp, 507 bp and 650 bp respectively. In some cases, unexpected PCR products were seen. In the quantitative RT-PCR trials described below, these various bands were

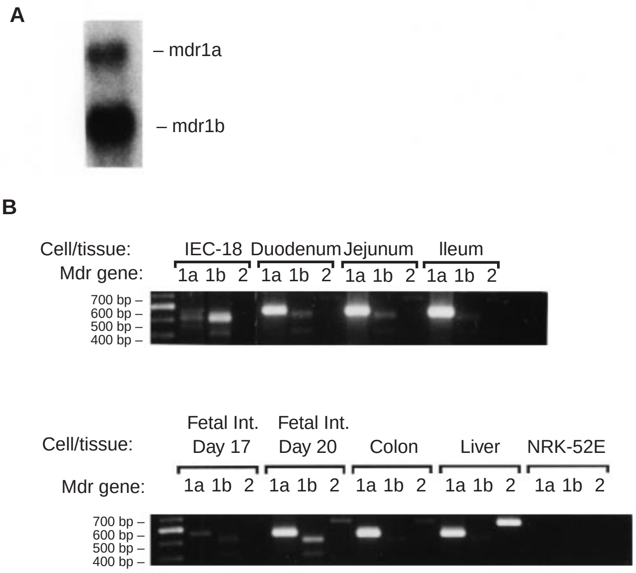

**Figure 3** RT/PCR detection of MDR gene expression. (**A**) Northern blot of RNA from IEC-18 cells hybridized with the generic MDR probe, 2B13-155. The mdr1a and mdr1b transcripts of 5.2 and 4.4 kb, respectively, were determined relative to the mobilities of the 28S and 18S ribosomal bands. (**B**) Ethidium bromide stained 2% agarose gels depicting products of MDR gene-specific PCR reactions. A 100-bp DNA ladder is loaded as a size marker. Gene-specific PCR amplifications from various cells and intestinal tissues. Whole tissue was used for RNA isolation from fetal intestine, small intestine, colon and liver observed inconsistently and were always less intense than the predicted product. Some of these bands were also observed in the negative control NRK-52E cells and were considered non-specific PCR products; others likely represent alternative-splicing in the 3′-untranslated region of MDR genes. Differences in 3′-untranslated region processing have been reported for MDR genes (Endicott et al, 1987; Van der Bliek et al, 1987) and are of unknown biological significance. In this study, only the predicted PCR product size, which is usually the dominant product, was considered.

In Figure 3B, the greater steady-state expression of mdr1b than mdr1a in IEC-18 cells is reflected in the RT-PCR assay. Mdr2 expression cannot be detected in IEC-18 cells. The relatively quantitative nature of the RT-PCR is further evidenced by MDR amplification of rat liver RNA, where the high levels of mdr1a and mdr2 relative to mdr1b observed is consistent with the previously documented pattern seen in liver (Brown et al, 1993; Lee et al, 1993). In contrast to the MDR expression profile of IEC-18 cells, whole tissue RNA preparations from adult rat small intestine and colon express high levels of mdr1a, low levels of mdr1b and still lower amounts of mdr2. The general patterns do not appear to vary across the duodenum, jejunum and ileum. Intermediate patterns of MDR expression are seen in fetal rat intestine. At day 17 of gestation, mdr1a and mdr1b are expressed in approximately equal amounts. By day 20, the adult pattern of MDR expression is beginning to emerge.

A quantitative RT-PCR assay was used to obtain numerical data on the relative expression of mdr1a and mdr1b across the proximal–distal and crypt–villus axes of adult intestine, and to determine whether the patterns of MDR expression seen in IEC-18 cells reflect those seen in undifferentiated crypt/fetal intestinal cells. Commonly used normalizing genes such as tubulin, actin,

 *© 1999 Cancer Research Campaign*

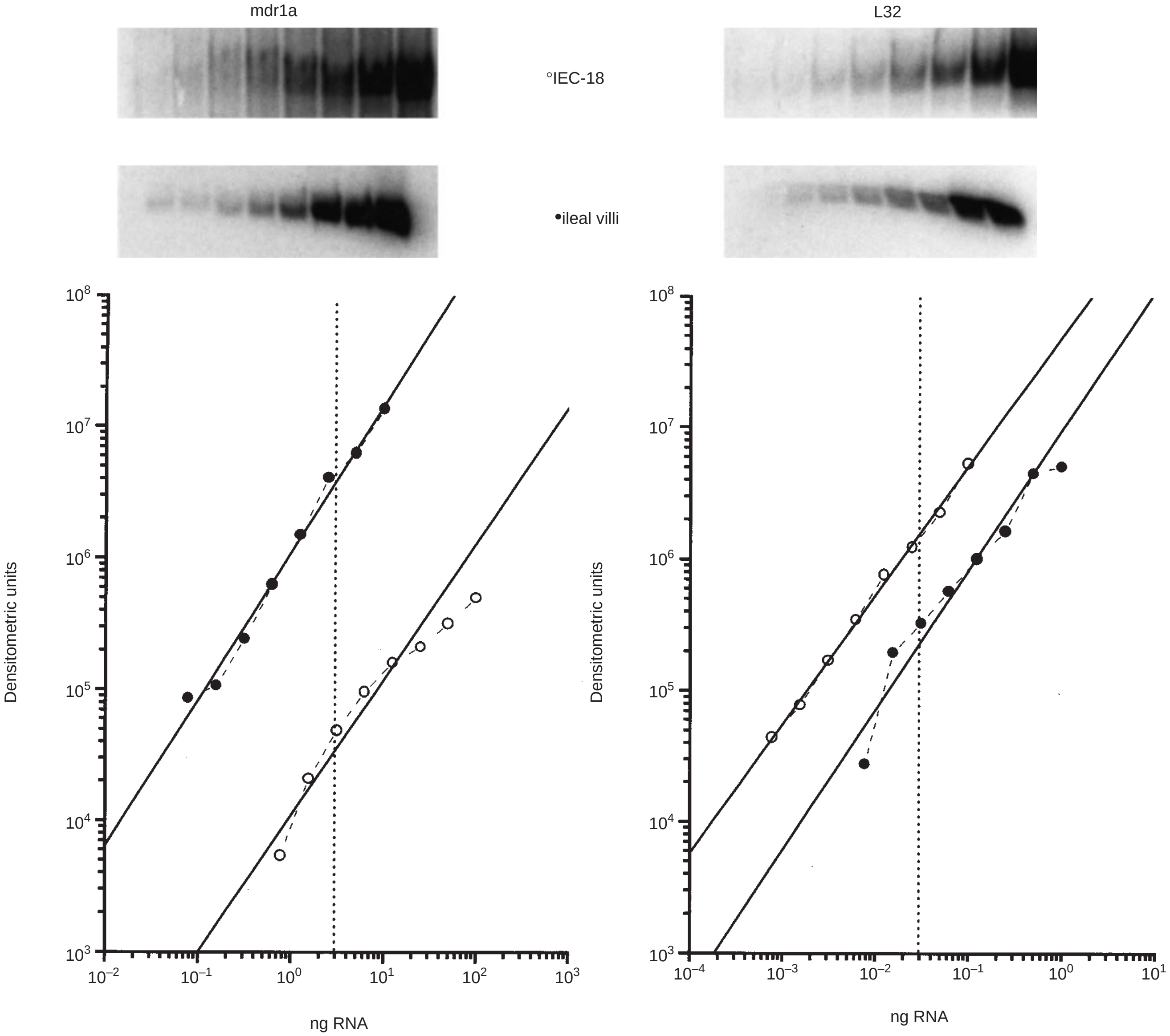

**Figure 4** Quantitative RT/PCR of mdr1a gene expression. Representative images and log–log plots of PCR products from eight serial 1:2 dilutions of IEC-18 and ileal villi cDNA. At the top are photographs of phosphorimager data set to linear ranges which optimize visualization of the bands. Amplifications of mdr1a and L32 are shown with the highest dilution beginning on the left. For log–log plots, raw phosphorimaging data of the above images are depicted with symbols and dashed lines, with the fitted linear regression superimposed in solid lines. Dotted vertical lines indicate the level of input RNA selected for comparison between samples glyceraldehyde-3-phosphate dehydrogenase and β2-microglobulin were all expressed at vastly different levels in small intestinal crypts and villi (not shown). Ribosomal L32 exhibited the least variation among samples and was used as an internal RNA standard to control for the actual amount of starting RNA, sample degradation and efficiency of the cDNA reaction. For quantitative RT-PCR, the amount of initiating cDNA which permits amplification in the exponential range had to be empirically determined for each gene in each cell type (see Materials and Methods). These values reflect the different levels of specific transcript in the various cells; for L32, the amounts of initiating cDNA required for each of the cell types are consistent with their relative expression of L32 as demonstrated by Northern blot analysis. Using ethidium bromide staining of ribosomal RNA bands for normalization, the relative expression levels of L32 were determined to be 7 times less in villi, 4 times less in small intestinal crypts, and 2 times more in fetal intestine as compared to IEC-18 cells and colonic crypts (data not shown). These values were factored in when using L32 as a normalizing standard.

Shown in Figure 4 are typical results from a quantitative RT-PCR experiment comparing mdr1a expression in IEC-18 cells and ileal villi. For all samples, mdr1a levels are compared at 3 ng of input RNA, which is within the exponential range of amplification. L32 levels were analysed at 30 pg and multiplied by 7 for intestinal villi, multiplied by 4 for small intestinal crypts, and divided by 2 for fetal intestine to correct for the average differential expression of L32. In Figure 4, the apparent 6.8-fold difference in L32 levels between IEC-18 cells and ileal villi reflects the true

*© 1999 Cancer Research Campaign*

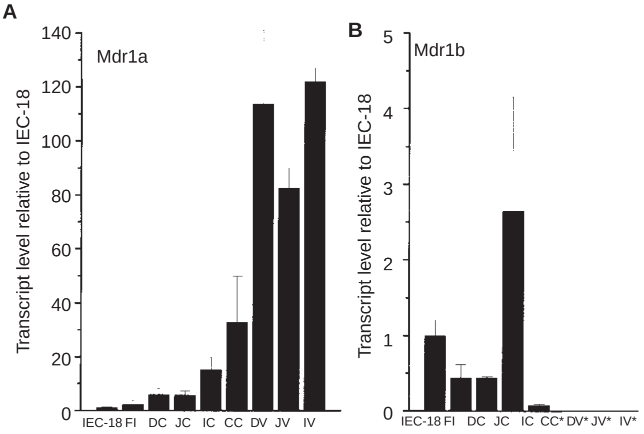

**Figure 5** Summary of relative MDR gene expression levels in intestinal cells. (**A**) Mdr1a transcript levels relative to IEC-18 in day 17 fetal intestine (FI), duodenal, jejunal, ileal and colonic crypts (DC, JC, IC, CC respectively) and duodenal, jejunal and ileal villi (DV, JV, IV respectively). (**B**) Mdr1b expression relative to IEC-18 in the same samples as panel **A**. *Samples where mdr1b expression was below the limits of reliable quantification using this assay. These data represents the mean and standard error derived from analysis of two separate isolations from each of three different rats

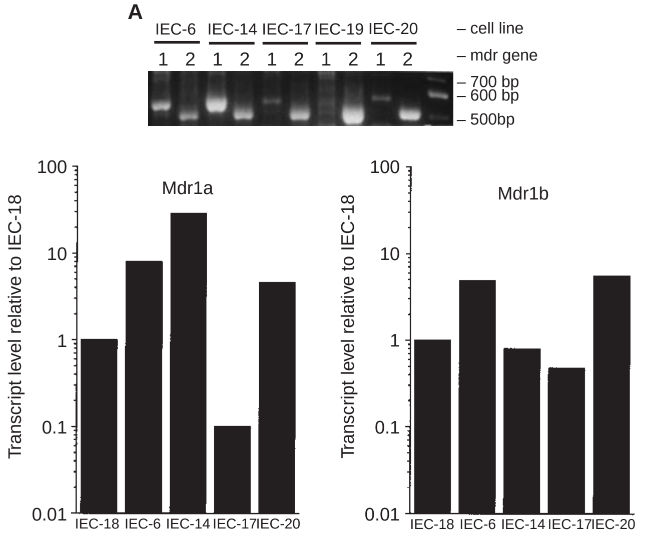

**Figure 6** MDR gene expression in IEC cells. (**A**) RT-PCR detection of mdr1a and mdr1b in a panel of five primitive rat intestinal epithelial cell lines. (**B**) Quantitative RT-PCR of MDR genes in IEC cells. The graph summarizes the expression of mdr1a and mdr1b in IEC cells, relative to IEC-18. Mdr1a levels in IEC-19 and mdr1b in IEC-20 were not determined in this experiment sevenfold difference in their steady-state expression of L32 as demonstrated by Northern probing. Thus, normalization of mdr1a densitometric units by corrected L32 densitometric units, $(3.6 \times 10^6)/(2.3 \times 10^5 \times 7) = 2.24$ and $(3.3 \times 10^4)/(1.5 \times 10^6) = 0.022$, results in a difference in mdr1a expression of $2.24/0.022 = 102$-fold between ileal villi and IEC-18 cells. Mdr1b levels were measured at the same time as mdr1a and L32, and analysed in an identical fashion, with mdr1b densitometric units being compared at 5 ng of input RNA across all samples.

Figure 5 summarizes the amounts of MDR expression in various tissues relative to that of IEC-18 cells. As seen in Figure 5A, mdr1a expression in IEC-18 cells is similar to that in fetal intestine, approximately 10 times less than small intestinal crypts and 100 times less than villi. In contrast, mdr1b levels in IEC-18 cells are on the same order of magnitude as fetal intestine and small intestinal crypts (Figure 5B). Levels of mdr1b transcript in small intestinal villi and colonic crypts were below the limits of reliable quantification in this assay. Along the crypt–villus axis there is clearly a predominance of mdr1a in villi, while both mdr1a and mdr1b are expressed in crypts. There do not appear to be large differences in MDR gene expression along the intestinal proximal–distal axis, although jejunal villi express slightly less mdr1a and jejunal crypts more mdr1b. MDR expression in colonic crypts parallels that of intestinal villi. From this analysis, it is apparent that there is a progression from mixed mdr1a and mdr1b expression to predominantly mdr1a as cells mature from crypts to villi in the small intestine. Although the mdr1b expression seen in fetal intestine may include a contribution from other cell types, IEC-18 cells appear to reflect the MDR expression profile of fetal intestine around day 17 of gestation.

### MDR gene expression in the IEC series

To determine whether the MDR expression pattern observed in IEC-18 cells could be generalized to other cells of the IEC series, mdr1a and mdr1b expression was analysed in the other five IEC cell lines – IEC-6, IEC-14, IEC-17, IEC-19 and IEC-20. As in IEC-18, RT-PCR analysis shows that mdr1b is highly expressed in these cell lines, in most cases in excess of mdr1a (Figure 6A). In a single trial of quantitative RT-PCR with the same RNA, these general patterns are confirmed, relative to the transcript levels in IEC-18 (Figure 6B). IEC-14 appears to be an exception, apparently expressing more mdr1a than mdr1b and in fact this cell line is unique from the others in that it was originally isolated as an epithelial clone growing out of a fibroblast culture (Quaroni et al, 1979). However, the magnitude of the differences in MDR gene expression seen in Figure 6B are on the order of that seen between IEC-18 and fetal or small intestinal crypt cells. Thus, the higher expression of mdr1a seen in IEC-14 is on the same order of magnitude seen in small intestinal crypts, rather than villi.

### DISCUSSION

MDR gene expression was characterized in IEC-18 cells in order to explore gene regulation in the undifferentiated intestinal epithelium. To identify promoter elements which may regulate gene expression in primitive intestinal cells, the hamster class I promoter was characterized in IEC-18 cells.

Deletion mapping of a previously established series of hamster pgp1 promoter constructs identified a putative repressor region between –299 and –254. Transcriptional repression in eukaryotes can occur through a number of mechanisms which range from the action of true silencers to interference with the action of transcriptional activators (for reviews see Herschbach and Johnson, 1993; Cowell, 1994). The inability of the repressor region to function on a heterologous promoter in a position and orientation independent manner indicates that this region does not contain a typical silencer. The repressor region may require interaction with elements in the pgp1 promoter not included in the present constructs. If so, this region would bind a factor which inhibits the

*© 1999 Cancer Research Campaign*

```
                                     NF-IL6
Ha-pgp1   –102 TCTAGAAATA  CAACCTGTTT CGCAATTGCT  CCTGCAATAA  TCCATGCA
Mu-mdr1a  –168 TCTACAAATC  CAACCTGTTT CGCAATTTCT  CCAGCAATAA  TACTTGAG
Hu-MDR1   –165 CCTGGAAATT  CAACCTGTTT CGCAGTTTCT  CGAGGAATCA  GCATTCAG

Mu-mdr1b  –131 CCTGGAAGTA  TCTCCTGTTT CGCAACCGCT  CCAGCTGCCC  ATGCCCAA
Ra-mdr1b  –129 CCTGGAAACC  ATCCCTATTT .GCAACCGCT  CCAGC.CTCC  TTGCCCAA
Ha-pgp2   –92  CCAGGAAGCG  CCTCCGGTTT GGTTTCGCAA  CCTCGCCTGC  .GGGCTCG
```

**Figure 7** Enhancer sequence alignment. The sequence of the hamster pgp1 enhancer from –102 to –52 is depicted, aligned with the available MDR gene promoters. There is significant conservation across the region, with the NF-IL6 binding site boxed and the 14 nucleotide stretch (Cohen et al, 1994) underlined function of a specific activator either by competing for DNA binding or blocking activation. A search through the NCBI transcription factor database failed to identify a match to any known promoter elements. However, this region does contain a stretch – GCAGAAGGCCAAGTGCA – which is identically conserved in the murine class 1 promoter (Hsu et al, 1990). Further studies demonstrating protein binding to this region and interaction with neighbouring enhancer regions will be required.

A region fulfilling the minimal requirements for an enhancer was identified between –100 and –52. This region was shown to enhance transcription in both forward and reverse orientations, from a heterologous promoter, specifically in IEC-18 cells. The lack of activity in SW620 (human colon carcinoma), RC3 (rat hepatoma) and AuxB1 (hamster ovary) indicates that this enhancer functions preferentially in primitive small intestinal epithelial cells. Located within this region is an enhancer activity and consensus NF-IL6 binding site which is conserved between murine mdr1a and mdr1b, human mdr1, and hamster pgp1 promoters (Yu et al, 1993; Combates et al, 1994) (Figure 7). In addition, an overlapping 14 nucleotide stretch – CAACCT-GTTTCGCA – is identically conserved between class I promoters from human, mouse and hamster (Hsu et al, 1990; Madden et al, 1993; Zastawny and Ling, 1993) (Figure 7). Deletion of this 14-bp element from the murine mdr1a promoter was shown to decrease reporter gene expression in NIH 3T3 fibroblasts and a DNAase I footprint was demonstrated in macrophage-like cell lines (Cohen et al, 1994). It remains to be determined whether the region exhibiting enhancer activity in IEC-18 cells corresponds to these same elements, however the enhancer identified here does appear to demonstrate specificity for undifferentiated intestinal cells.

MDR gene expression in the intestinal epithelium has been reported by numerous investigators in human (Fojo et al, 1987; Thiebaut et al, 1987), mouse (Croop et al, 1989; Buschman et al, 1992), hamster (Mukhopadhyay et al, 1988; Bradley et al, 1990) and rat (Trezise and Buchwald, 1991). These studies have all identified the class I gene (MDR1, mdr1a and pgp1 respectively) as the only isoform in the intestinal epithelium. Since Northern probing unexpectedly revealed a greater expression of mdr1b than mdr1a in IEC-18 cells, gene-specific RT-PCR assays were used to analyse MDR transcripts in rat intestine and validate the use of IEC-18 as a model.

The results confirm that the class I transcript is the predominant MDR gene expressed in the adult intestine. However, lower levels of both MDR class II and class III transcripts were also detected. Although Furuya et al (1994) have reported the expression of class III transcript in the rat intestine, detection of class II transcript in the intestinal epithelium is a novel finding. Class II transcript has not been detected in previous studies due to the use of techniques with insufficient sensitivity, or because of experimental design that implicity assumes only class I transcript is expressed in the intestine.

The results presented here reveal that mdr1a transcript levels are highest in the small intestine with lower levels in the colon. This is consistent with the findings of Bremer et al (1992) and Trezise and Buchwald (1991), who employed quantitative RT-PCR and RNA in situ hybridization techniques respectively. Using isoform-specific monoclonal antibodies in the hamster digestive tract, Bradley et al (1990) identified the highest levels of pgp1 expression in the caecum and proximal colon with barely detectable levels in the rest of the intestine. The discrepancy with the Bradley findings are most likely due to differences between transcript and protein expression. At present, the regulation of mdr1a expression in normal tissue is poorly understood, but is likely to occur at many levels including gene amplification, transcription and translation.

Consistent with the findings of Tresize and Buchwald (1991), variations in mdr1a level along the horizontal axis of the small intestine were not large. However, expression across the crypt–villus axis varied considerably, with villi expressing at least an order of magnitude more than crypts or day 17 fetal intestine. In contrast, mdr1b was expressed preferentially in small intestinal crypts and fetal intestine, with levels in villi below the limits of quantification. Thus, it appears that there is a conversion from mixed mdr1a and mdr1b to predominantly mdr1a expression as cells mature along the crypt–villus axis.

If the expression of MDR genes in fetal intestine reflects the different function of the prenatal digestive tract, which is to absorb amniotic fluid rather than digest food and excrete toxins, then analysis of P-glycoprotein isoforms in the undifferentiated intestine may shed light on their different physiologic functions. It is likely that different functions of the MDR1 gene in humans are divided between mdr1a and mdr1b in rodents. The observed predominance of mdr1a in intestinal villi is consistent with a protective function in the gut and this is borne out in mice with a disrupted mdr1a gene (Borst and Schinkel, 1997). Mdr1a was shown to be important for maintenance of the blood-brain barrier and in the gut, unopposed reabsorption resulted in reduced clearance of drugs dependent on P-glycoprotein transport. Significantly, deletion of mdr1a expression resulted in an increase in mdr1b transcript in liver and kidney, but expression in the intestinal epithelium was not assessed.

The mdr1a and mdr1b expression pattern of IEC cells was shown to be much more similar to fetal or small intestinal crypt cells than villus cells. Fetal intestinal cells and adult crypts cells are thought to be phenotypically similar (Quaroni, 1986), and cells of the IEC series are thought to represent primitive, undifferentiated intestinal epithelial cells based on their surface antigen profile and their developmental potential (Quaroni et al, 1979; Quaroni and Isselbacher, 1981; Quaroni, 1985). The findings of this study lend further support to this concept. The pattern of MDR transcript expression in IEC cells therefore, make them an appropriate model for the analysis of MDR gene regulation in undifferentiated intestinal epithelial cells.

*© 1999 Cancer Research Campaign*

In this report, MDR gene expression in IEC-18 cells was characterized as a model system with which to explore transcriptional regulation in the undifferentiated intestinal epithelium. Two unique contributions to this field are described. One, the initial identification of an undifferentiated cell-specific enhancer region in the hamster mdr1a promoter and the other, detection of mdr1b expression in primitive intestinal cell types. Intriguingly, the enhancer was identified in the promoter of mdr1a, a transcript which is more highly expressed in differentiated intestinal cells. It remains to be determined whether this is due to relatively increased activity of an mdr1a repressor, or decreased mdr1a transcript stability in undifferentiated cells. This work provides a basis for such future studies and to discover new transcription factors involved in determining cellular identity during intestinal development, differentiation and tumorigenesis.

### ACKNOWLEDGEMENTS

We thank Dr Jorge Filmus and Dr Danielle Cano-Gauci for critical reading of the manuscript; Barbara Choo for excellent technical assistance. This work was supported by a Medical Research Council of Canada studentship to ML and a Terry Fox Program Project Grant to RNB.

### REFERENCES

(1993) *Current Protocols in Molecular Biology*, Vol. 1. John Wiley & Sons: New York

Asp NG, Gudmand-Hoyer E, Andersen B, Berg NO and Dahlqvist A (1975) Distribution of disaccharidases, alkaline phosphatase, and some intracellular enzymes along the human small intestine. *Scand J Gastroenterol* **10**: 647–651

Borst P and Schinkel AH (1997) Genetic dissection of the function of mammalian P-glycoproteins. *Trends Genet* **13**:

Bradley G, Georges E and Ling V (1990) Sex-dependent and independent expression of the P-glycoprotein isoforms in Chinese hamster. *J Cell Physiol* **145**: 398–408

Bremer S, Hoof T, Wilke M, Busche R, Scholte B, Riordan JR, Maass G and Tummler B (1992) Quantitative expression patterns of multidrug-resistance P-glycoprotein (MDR1) and differentially spliced cystic-fibrosis transmembrane-conductance regulator mRNA transcripts in human epithelia. *Eur J Biochem* **206**: 137–149

Brown PC, Thorgeirsson SS and Silverman JA (1993) Cloning and regulation of the rat mdr2 gene. *Nucleic Acids Res* **21**: 3885–3891

Buschman E, Arceci RJ, Croop JM, Che M, Arias IM, Housman DE and Gros P (1992) mdr2 encodes P-glycoprotein expressed in the bile canalicular membrane as determined by isoform-specific antibodies. *J Biol Chem* **267**: 18093–18099

Cano-Gauci DF, Lualdi JC, Ouellette AJ, Brady G, Iscove NN and Buick RN (1993) In vitro cDNA amplification from individual intestinal crypts: a novel approach to the study of differential gene expression along the crypt-villus axis. *Exp Cell Res* **208**: 344–349

Chomczynski P and Sacchi N (1987) Single-step method of RNA isolation by acid guanidinium thiocyanate phenol chloroform extraction. *Anal Biochem* **162**: 156–159

Cirillo LA, Emerson JA, Vacher J and Tyner AL (1995) Developmental regulation of alpha-fetoprotein expression in intestinal epithelial cells of transgenic mice. *Dev Biol* **168**: 395–405

Cohen D, Yu L, Rzepka R and Horwitz SB (1994) Identification of two nuclear protein binding sites and their role in the regulation of the murine multidrug resistance mdr1a promoter. *DNA Cell Biol* **13**: 641–649

Cohn SM, Simon TC, Roth KA, Birkenmeier EH and Gordon JI (1992) Use of transgenic mice to map cis-acting elements in the intestinal fatty acid binding protein gene (Fabpi) that control its cell lineage-specific and regional patterns of expression along the duodenal-colonic and crypt-villus axes of the gut epithelium. *J Cell Biol* **119**: 27–44

Combates NJ, Rzepka RW, Chen Y-NP and Cohen D (1994) NF-IL6, a member of the C/EBP family of transcription factors, binds and *trans*-activates the human MDR1 gene promoter. *J Biol Chem* **269**: 29715–29719

Cowell IG (1994) Repression versus activation in the control of gene transcription. *Trends Biochem Sci* **19**: 38–42

Croop JM, Raymond M, Haber D, Devault A, Arceci RJ, Gros P and Housman DE (1989) The three mouse multidrug resistance (mdr) genes are expressed in a tissue-specific manner in normal mouse tissues. *Mol Cell Biol* **9**: 1346–1350

Deren JJ (1987) Development of intestinal structure and function. In *Handbook of Physiology, Vol. III - Intestinal absorption*. American Physiological Society: Washington DC.

Deuchars KL, Duthie M and Ling V (1992) Identification of distinct P-glycoprotein gene sequences in rat. *Biochim Biophys Acta* **1130**: 157–165

Dudov KP and Perry RP (1984) The gene family encoding the mouse ribosomal protein L32 contains a uniquely expressed intron-containing gene and an unmutated processed gene. *Cell* **37**: 457–468

Endicott JA, Juranka PF, Sarangi F, Gerlach JH, Deuchars KL and Ling V (1987) Simultaneous expression of two P-glycoprotein genes in drug-sensitive Chinese hamster ovary cells. *Mol Cell Biol* **7**: 4075–4081

Fojo AT, Ueda K, Slamon DJ, Poplack DG, Gottesman MM and Pastan I (1987) Expression of a multidrug-resistance gene in human tumors and tissues. *Proc Natl Acad Sci USA* **84**: 265–269

Furuya KN, Gebhardt R, Schuetz EG and Schuetz JD (1994) Isolation of rat pgp3 cDNA: evidence for gender and zonal regulation of expression in the liver. *Biochim Biophys Acta* **1219**: 636–644

Gatmaitan ZC and Arias IM (1993) Structure and function of P-glycoprotein in normal liver and small intestine. *Adv Pharmacol* **24**: 77–97

Gottesman MM and Pastan I (1993) Biochemistry of multidrug resistance mediated by the multidrug transporter. *Annu Rev Biochem* **62**: 385–427

Gottesman MM, Hrycyna CA, Schoenlein PV, Germann UA and Pastan I (1995) Genetic analysis of the multidrug transporter. *Annu Rev Genet* **29**: 607–649

Gottesman MM, Pastan I and Ambudkar SV (1996) P-glycoprotein and multidrug resistance. *Curr Opin Genet Dev* **6**: 610–617

Herschbach BM and Johnson AD (1993) Transcriptional repression in eukaryotes. *Annu Rev Cell Biol* **9**: 479–509

Hsu SI, Cohen D, Kirschner LS, Lothstein L, Hartstein M and Horwitz SB (1990) Structural analysis of the mouse mdr1a (P-glycoprotein) promoter reveals the basis for differential transcript heterogeneity in multidrug-resistant J774.2 cells [published erratum appears in *Mol Cell Biol* 1990 **10**: 6101]. *Mol Cell Biol* **10**: 3596–3606

Hu Y, Kazenwadel J and James R (1993) Isolation and characterization of the murine homeobox gene Cdx-1. Regulation of expression in intestinal epithelial cells. *J Biol Chem* **268**: 27214–27225

Koh J, Sferra TJ and Collins FS (1993) Characterization of the cystic fibrosis transmembrane conductance regulator promoter region. Chromatin context and tissue-specificity. *J Biol Chem* **268**: 15912–15921

Lee CH, Bradley G, Zhang JT and Ling V (1993) Differential expression of P-glycoprotein genes in primary rat hepatocyte culture. *J Cell Physiol* **157**: 392–402

McDonald RA, Matthews RP, Idzerda RL and McKnight GS (1995) Basal expression of the cystic fibrosis transmembrane conductance regulator gene is dependent on protein kinase A activity. *Proc Natl Acad Sci USA* **92**: 7560–7564

Madden MJ, Morrow CS, Nakagawa M, Goldsmith ME, Fairchild CR and Cowan KH (1993) Identification of 5′ and 3′ sequences involved in the regulation of transcription of the human mdr1 gene in vivo. *J Biol Chem* **268**: 8290–8297

Mukhopadhyay T, Batsakis JG and Kuo MT (1988) Expression of the mdr (P-glycoprotein) gene in Chinese hamster digestive tracts. *J Natl Cancer Inst* **80**: 269–275

Murphy LD, Herzog CE, Rudick JB, Fojo AT and Bates SE (1990) Use of the polymerase chain reaction in the quantitation of mdr-1 gene expression. *Biochemistry* **29**: 10351–10356

Potten CS and Loeffler M (1990) Stem cells: attributes, cycles, spirals, pitfalls and uncertainties. Lessons for and from the crypt. *Development* **110**: 1001–1020

Quaroni A (1985) Development of fetal rat intestine in organ and monolayer culture. *J Cell Biol* **100**: 1611–1622

Quaroni A (1986) Fetal characteristics of small intestinal crypt cells. *Proc Natl Acad Sci USA* **83**: 1723–1727

Quaroni A and Isselbacher KJ. (1981) Cytotoxic effects and metabolism of benzo[a]pyrene and 7,12-dimethylbenz[a]anthracene in duodenal and ileal epithelial cell cultures. *J Natl Cancer Inst* **67**: 1353–1362

Quaroni A, Wands J, Trelstad RL and Isselbacher KJ (1979) Epithelioid cell cultures from rat small intestine. Characterization by morphologic and immunologic criteria. *J Cell Biol* **80**: 248–265

Rajchel A, Chan YL and Wool IG (1988) The primary structure of rat ribosomal protein L32. *Nucleic Acids Res* **16**: 2347

 *© 1999 Cancer Research Campaign*

Ruetz S and Gros P (1994) Phosphatidylcholine translocase: a physiological role for the mdr2 gene. *Cell* **77**: 1071–1081

Schrenk D, Gant TW, Preisegger K-H, Silverman JA, Marino PA and Thorgeirsson SS (1992) Induction of multidrug resistance gene expression during cholestasis in rats and non-human primates. *Hepatology* **17**: 854–860

Silverman JA and Hill BA (1995) Characterization of the basal and carcinogen regulatory elements of the rat mdr1b promoter. *Mol Carcinog* **13**: 50–59

Silverman JA, Raunio H, Gant TW and Thorgeirsson SS (1991) Cloning and characterization of a member of the rat multidrug resistance (*mdr*) gene family. *Gene* **106**: 229–236

Smit JJ, Mol CA, van Deemter L, Wagenaar E, Schinkel AH and Borst P (1995) Characterization of the promoter region of the human MDR3 P-glycoprotein gene. *Biochim Biophys Acta* **1261**: 44–56

Song R, Ikeguchi M, Zhou G and Kuo MT (1995) Identification and characterization of a hepatoma cell-specific enhancer in the mouse multidrug resistance mdr1b promoter. *J Biol Chem* **270**: 25468–25474

Suh E, Chen L, Taylor J and Traber PG (1994) A homeodomain protein related to caudal regulates intestine-specific gene transcription. *Mol Cell Biol* **14**: 7340–7351

Thiebaut F, Tsuruo T, Hamada H, Gottesman MM, Pastan I and Willingham MC (1987) Cellular localization of the multidrug-resistance gene product P-glycoprotein in normal human tissues. *Proc Natl Acad Sci USA* **84**: 7735–7738

Thorgeirsson SS, Gant TW and Silverman JA (1994) Transcriptional regulation of multidrug resistance gene expression. *Cancer Treat Res* **73**: 57–68

Traber PG (1990) Regulation of sucrase–isomaltase gene expression along the crypt–villus axis of rat small intestine. *Biochem Biophys Res Commun* **173**: 765–773

Traber PG (1994) Differentiation of intestinal epithelial cells: lesions from the study of intestine-specific gene expression. *J Lab Clin Med* **123**: 467–477

Trezise AE and Buchwald M (1991) In vivo cell-specific expression of the cystic fibrosis transmembrane conductance regulator. *Nature* **353**: 434–437

Trier JS and Moxey PC (1979) Morphogenesis of the small intestine during fetal development. *Ciba Found Symp* **3**: 29

Tyner AL, Godbout R, Compton RS and Tilghman SM (1990) The ontogeny of alpha-fetoprotein gene expression in the mouse gastrointestinal tract. *J Cell Biol* **110**: 915–927

Van der Bliek AM, Baas F, Ten Houte de Lange T, Kooiman PM, Van der Velde-Koerts T and Borst P (1987) The human mdr3 gene encodes a novel P-glycoprotein homologue and gives rise to alternatively spliced mRNAs in liver. *Embo J* **6**: 3325–3331

Yu LY, Cohen D, Piekarz RL and Horwitz SB (1993) Three distinct nuclear protein binding sites in the promoter of the murine multidrug resistance *mdr1b* gene.

*J Biol Chem* **268**: 7520–7526

Zastawny RL and Ling V (1993) Structural and functional analysis of 5′ flanking and intron 1 sequences of the hamster P-glycoprotein pgp1 and pgp2 genes. *Biochim Biophys Acta* **1173**: 303–313

*© 1999 Cancer Research Campaign* 

## References

[PDF_01058] Asp N. G., Gudmand-Höyer E., Andersen B., Berg N. O., Dahlqvist A. (1975). Distribution of disaccharidases, alkaline phosphatase, and some intracellular enzymes along the human small intestine.. Scand J Gastroenterol.

[PDF_01064] Bradley G., Georges E., Ling V. (1990). Sex-dependent and independent expression of the P-glycoprotein isoforms in Chinese hamster.. J Cell Physiol.

[PDF_01067] Bremer S., Hoof T., Wilke M., Busche R., Scholte B., Riordan J. R., Maass G., Tümmler B. (1992). Quantitative expression patterns of multidrug-resistance P-glycoprotein (MDR1) and differentially spliced cystic-fibrosis transmembrane-conductance regulator mRNA transcripts in human epithelia.. Eur J Biochem.

[PDF_01072] Brown P. C., Thorgeirsson S. S., Silverman J. A. (1993). Cloning and regulation of the rat mdr2 gene.. Nucleic Acids Res.

[PDF_01074] Buschman E., Arceci R. J., Croop J. M., Che M., Arias I. M., Housman D. E., Gros P. (1992). mdr2 encodes P-glycoprotein expressed in the bile canalicular membrane as determined by isoform-specific antibodies.. J Biol Chem.

[PDF_01078] Cano-Gauci D. F., Lualdi J. C., Ouellette A. J., Brady G., Iscove N. N., Buick R. N. (1993). In vitro cDNA amplification from individual intestinal crypts: a novel approach to the study of differential gene expression along the crypt-villus axis.. Exp Cell Res.

[PDF_01082] Chomczynski P., Sacchi N. (1987). Single-step method of RNA isolation by acid guanidinium thiocyanate-phenol-chloroform extraction.. Anal Biochem.

[PDF_01085] Cirillo L. A., Emerson J. A., Vacher J., Tyner A. L. (1995). Developmental regulation of alpha-fetoprotein expression in intestinal epithelial cells of transgenic mice.. Dev Biol.

[PDF_01088] Cohen D., Yu L., Rzepka R., Horwitz S. B. (1994). Identification of two nuclear protein binding sites and their role in the regulation of the murine multidrug resistance mdr1a promoter.. DNA Cell Biol.

[PDF_01091] Cohn S. M., Simon T. C., Roth K. A., Birkenmeier E. H., Gordon J. I. (1992). Use of transgenic mice to map cis-acting elements in the intestinal fatty acid binding protein gene (Fabpi) that control its cell lineage-specific and regional patterns of expression along the duodenal-colonic and crypt-villus axes of the gut epithelium.. J Cell Biol.

[PDF_01096] Combates N. J., Rzepka R. W., Chen Y. N., Cohen D. (1994). NF-IL6, a member of the C/EBP family of transcription factors, binds and trans-activates the human MDR1 gene promoter.. J Biol Chem.

[PDF_01099] Cowell I. G. (1994). Repression versus activation in the control of gene transcription.. Trends Biochem Sci.

[PDF_01101] Croop J. M., Raymond M., Haber D., Devault A., Arceci R. J., Gros P., Housman D. E. (1989). The three mouse multidrug resistance (mdr) genes are expressed in a tissue-specific manner in normal mouse tissues.. Mol Cell Biol.

[PDF_01107] Deuchars K. L., Duthie M., Ling V. (1992). Identification of distinct P-glycoprotein gene sequences in rat.. Biochim Biophys Acta.

[PDF_01109] Dudov K. P., Perry R. P. (1984). The gene family encoding the mouse ribosomal protein L32 contains a uniquely expressed intron-containing gene and an unmutated processed gene.. Cell.

[PDF_01112] Endicott J. A., Juranka P. F., Sarangi F., Gerlach J. H., Deuchars K. L., Ling V. (1987). Simultaneous expression of two P-glycoprotein genes in drug-sensitive Chinese hamster ovary cells.. Mol Cell Biol.

[PDF_01115] Fojo A. T., Ueda K., Slamon D. J., Poplack D. G., Gottesman M. M., Pastan I. (1987). Expression of a multidrug-resistance gene in human tumors and tissues.. Proc Natl Acad Sci U S A.

[PDF_01118] Furuya K. N., Gebhardt R., Schuetz E. G., Schuetz J. D. (1994). Isolation of rat pgp3 cDNA: evidence for gender and zonal regulation of expression in the liver.. Biochim Biophys Acta.

[PDF_01121] Gatmaitan Z. C., Arias I. M. (1993). Structure and function of P-glycoprotein in normal liver and small intestine.. Adv Pharmacol.

[PDF_01125] Gottesman M. M., Hrycyna C. A., Schoenlein P. V., Germann U. A., Pastan I. (1995). Genetic analysis of the multidrug transporter.. Annu Rev Genet.

[PDF_01127] Gottesman M. M., Pastan I., Ambudkar S. V. (1996). P-glycoprotein and multidrug resistance.. Curr Opin Genet Dev.

[PDF_01123] Gottesman M. M., Pastan I. (1993). Biochemistry of multidrug resistance mediated by the multidrug transporter.. Annu Rev Biochem.

[PDF_01129] Herschbach B. M., Johnson A. D. (1993). Transcriptional repression in eukaryotes.. Annu Rev Cell Biol.

[PDF_01131] Hsu S. I., Cohen D., Kirschner L. S., Lothstein L., Hartstein M., Horwitz S. B. (1990). Structural analysis of the mouse mdr1a (P-glycoprotein) promoter reveals the basis for differential transcript heterogeneity in multidrug-resistant J774.2 cells.. Mol Cell Biol.

[PDF_01136] Hu Y., Kazenwadel J., James R. (1993). Isolation and characterization of the murine homeobox gene Cdx-1. Regulation of expression in intestinal epithelial cells.. J Biol Chem.

[PDF_01139] Koh J., Sferra T. J., Collins F. S. (1993). Characterization of the cystic fibrosis transmembrane conductance regulator promoter region. Chromatin context and tissue-specificity.. J Biol Chem.

[PDF_01142] Lee C. H., Bradley G., Zhang J. T., Ling V. (1993). Differential expression of P-glycoprotein genes in primary rat hepatocyte culture.. J Cell Physiol.

[PDF_01149] Madden M. J., Morrow C. S., Nakagawa M., Goldsmith M. E., Fairchild C. R., Cowan K. H. (1993). Identification of 5' and 3' sequences involved in the regulation of transcription of the human mdr1 gene in vivo.. J Biol Chem.

[PDF_01145] McDonald R. A., Matthews R. P., Idzerda R. L., McKnight G. S. (1995). Basal expression of the cystic fibrosis transmembrane conductance regulator gene is dependent on protein kinase A activity.. Proc Natl Acad Sci U S A.

[PDF_01152] Mukhopadhyay T., Batsakis J. G., Kuo M. T. (1988). Expression of the mdr (P-glycoprotein) gene in Chinese hamster digestive tracts.. J Natl Cancer Inst.

[PDF_01155] Murphy L. D., Herzog C. E., Rudick J. B., Fojo A. T., Bates S. E. (1990). Use of the polymerase chain reaction in the quantitation of mdr-1 gene expression.. Biochemistry.

[PDF_01158] Potten C. S., Loeffler M. (1990). Stem cells: attributes, cycles, spirals, pitfalls and uncertainties. Lessons for and from the crypt.. Development.

[PDF_01161] Quaroni A. (1985). Development of fetal rat intestine in organ and monolayer culture.. J Cell Biol.

[PDF_01163] Quaroni A. (1986). Fetal characteristics of small intestinal crypt cells.. Proc Natl Acad Sci U S A.

[PDF_01165] Quaroni A., Isselbacher K. J. (1981). Cytotoxic effects and metabolism of benzo[a]pyrene and 7,12-dimethylbenz[a]anthracene in duodenal and ileal epithelial cell cultures.. J Natl Cancer Inst.

[PDF_01168] Quaroni A., Wands J., Trelstad R. L., Isselbacher K. J. (1979). Epithelioid cell cultures from rat small intestine. Characterization by morphologic and immunologic criteria.. J Cell Biol.

[PDF_01171] Rajchel A., Chan Y. L., Wool I. G. (1988). The primary structure of rat ribosomal protein L32.. Nucleic Acids Res.

[PDF_01175] Ruetz S., Gros P. (1994). Phosphatidylcholine translocase: a physiological role for the mdr2 gene.. Cell.

[PDF_01177] Schrenk D., Gant T. W., Preisegger K. H., Silverman J. A., Marino P. A., Thorgeirsson S. S. (1993). Induction of multidrug resistance gene expression during cholestasis in rats and nonhuman primates.. Hepatology.

[PDF_01180] Silverman J. A., Hill B. A. (1995). Characterization of the basal and carcinogen regulatory elements of the rat mdr1b promoter.. Mol Carcinog.

[PDF_01182] Silverman J. A., Raunio H., Gant T. W., Thorgeirsson S. S. (1991). Cloning and characterization of a member of the rat multidrug resistance (mdr) gene family.. Gene.

[PDF_01185] Smit J. J., Mol C. A., van Deemter L., Wagenaar E., Schinkel A. H., Borst P. (1995). Characterization of the promoter region of the human MDR3 P-glycoprotein gene.. Biochim Biophys Acta.

[PDF_01188] Song R., Ikeguchi M., Zhou G., Kuo M. T. (1995). Identification and characterization of a hepatoma cell-specific enhancer in the mouse multidrug resistance mdr1b promoter.. J Biol Chem.

[PDF_01191] Suh E., Chen L., Taylor J., Traber P. G. (1994). A homeodomain protein related to caudal regulates intestine-specific gene transcription.. Mol Cell Biol.

[PDF_01194] Thiebaut F., Tsuruo T., Hamada H., Gottesman M. M., Pastan I., Willingham M. C. (1987). Cellular localization of the multidrug-resistance gene product P-glycoprotein in normal human tissues.. Proc Natl Acad Sci U S A.

[PDF_01197] Thorgeirsson S. S., Gant T. W., Silverman J. A. (1994). Transcriptional regulation of multidrug resistance gene expression.. Cancer Treat Res.

[PDF_01202] Traber P. G. (1994). Differentiation of intestinal epithelial cells: lessons from the study of intestine-specific gene expression.. J Lab Clin Med.

[PDF_01199] Traber P. G. (1990). Regulation of sucrase-isomaltase gene expression along the crypt-villus axis of rat small intestine.. Biochem Biophys Res Commun.

[PDF_01204] Trezise A. E., Buchwald M. (1991). In vivo cell-specific expression of the cystic fibrosis transmembrane conductance regulator.. Nature.

[PDF_01208] Tyner A. L., Godbout R., Compton R. S., Tilghman S. M. (1990). The ontogeny of alpha-fetoprotein gene expression in the mouse gastrointestinal tract.. J Cell Biol.

[PDF_01212] Van der Bliek A. M., Baas F., Ten Houte de Lange T., Kooiman P. M., Van der Velde-Koerts T., Borst P. (1987). The human mdr3 gene encodes a novel P-glycoprotein homologue and gives rise to alternatively spliced mRNAs in liver.. EMBO J.

[PDF_01215] Yu L., Cohen D., Piekarz R. L., Horwitz S. B. (1993). Three distinct nuclear protein binding sites in the promoter of the murine multidrug resistance mdr1b gene.. J Biol Chem.

[PDF_01218] Zastawny R. L., Ling V. (1993). Structural and functional analysis of 5' flanking and intron 1 sequences of the hamster P-glycoprotein pgp1 and pgp2 genes.. Biochim Biophys Acta.

